# A Longitudinal Assessment of Endometriosis Patients Prescribed Cannabis‐Based Medicinal Products: A Case Series From the UK Medical Cannabis Registry

**DOI:** 10.1111/ajo.70078

**Published:** 2025-11-26

**Authors:** Sara Getter, Simon Erridge, John Warner‐Levy, Evonne Clarke, Katy McLachlan, Ross Coomber, Shelley Barnes, Alia Darweish Medniuk, Rahul Guru, Wendy Holden, Mohammed Sajad, Robert Searle, Azfer Usmani, Sanjay Varma, James J. Rucker, Michael Platt, Mikael H. Sodergren

**Affiliations:** ^1^ Medical Cannabis Research Group Imperial College London London UK; ^2^ Curaleaf Clinic London UK; ^3^ St. George's Hospital NHS Trust London UK; ^4^ North Bristol NHS Trust Bristol UK; ^5^ Cardiff and Vale University Health Board Cardiff UK; ^6^ Department of Psychological Medicine Kings College London London UK; ^7^ South London & Maudsley NHS Foundation Trust London UK

**Keywords:** cannabidiol, cannabis, endometriosis, pain, tetrahydrocannabinol

## Abstract

**Background:**

Although there is growing evidence supporting the use of cannabis‐based medicinal products (CBMPs) for the management of chronic pain, there is a paucity of data on their effect on endometriosis‐associated chronic pain.

**Aims:**

This study aimed to perform an analysis of pain‐specific and general health‐related quality of life (HRQoL) outcomes for patients with endometriosis‐associated chronic pain treated with CBMPs.

**Materials and Methods:**

Primary outcomes included changes in patient‐reported outcome measures (PrOMs) from baseline to 1, 3, 6, 12 and 18 months. A repeated measures ANOVA was applied to assess changes in PrOMs at 1 to 18 months from baseline. Secondary outcomes included incidence and frequency of adverse events (AEs).

**Results:**

Sixty‐three patients met inclusion criteria. Initiation of CBMPs was associated with improvements in all pain‐specific PrOMs from baseline to 18 months (*p* < 0.050). EQ‐5D‐5L index value showed improvements between baseline and all months (*p* < 0.050). Anxiety and sleep quality PrOMs showed improvements from baseline to 18 months (*p* < 0.050). Minimal clinically significant differences (11%–37%), moderately important improvements (5%–22%) and substantial improvements (0%–11%) were observed in the Brief Pain Inventory (BPI) and pain severity visual analogue scale. Sixty‐two adverse events were reported by 16 (25.40%) participants.

**Conclusions:**

This study observed an association between CBMP treatment and improvements in pain and HRQoL in patients with endometriosis. Causality cannot be inferred due to the nature of this observational study; however, these findings provide complementary evidence for the development of randomised controlled trials to assess the efficacy of CBMPs for endometriosis‐associated chronic pain.

## 1 Introduction

Endometriosis affects approximately 176 million women globally [1]. It is characterised by growth of endometrial‐like tissue outside the uterus, causing symptoms including abdominal cramps, dysmenorrhea, infertility and chronic pelvic pain, impairing quality of life [1]. Current treatment options, such as nonsteroidal anti‐inflammatory drugs, offer limited pain relief and can have gastrointestinal, cardiovascular and renal side effects [2, 3].

Cannabis‐based medicinal products (CBMPs) that contain phytocannabinoids, such as (−)‐trans‐Δ9‐tetrahydrocannabinol (Δ9‐THC) and cannabidiol (CBD), have been identified with potential to manage endometriosis‐related pain [4]. Δ9‐THC is a partial agonist of cannabinoid receptor type 1 (CB1R), which modulates neurotransmitter release [4]. CBD primarily acts by inhibiting anandamide breakdown, an endogenous cannabinoid receptor agonist [2]. CBD is also a negative allosteric modulator of CB1R [2]. CBD is an inhibitor of transient receptor potential vanilloid 1 (TRPV1) signalling at high doses, reducing the intensity of nociceptive signals transmitted by TRPV1 [4]. Moreover, CBD is a partial agonist of 5‐hydroxytryptamine 1A, which may have a complementary role in modifying pain perception [5]. In a rat model of acute inflammation, a CB2 receptor agonist, GW405833, reduced pro‐inflammatory cytokine release, such as interleukin (IL)‐1β and tumour necrosis factor (TNF)‐α [5]. In a rat model of endometriosis, injection with 5 mg/kg CBD resulted in a reduction in TNF‐α, endometriotic implant surface area, IL‐6 and total oxidant status [6].

While there is growing research on CBMPs in chronic pain treatment [7], data on endometriosis‐associated chronic pain remain scarce. A meta‐analysis on chronic non‐cancer and cancer pain concluded that non‐inhaled CBMPs lead to modest enhancements in pain alleviation, sleep quality and physical capabilities [8]. However, this analysis only included one randomised controlled trial (RCT) on endometriosis‐associated chronic pain. The primary aim of this study is to examine the observed changes in pain‐specific and other patient‐reported outcome measures (PrOMs) related to other domains of health‐related quality of life (HRQoL) in people with endometriosis‐associated chronic pain treated with CBMPs.

## 2 Materials and Methods

### 2.1 Study Design and Participants

This is a prospective case series from the UK Medical Cannabis Registry (UKMCR) where endometriosis was the primary indication for treatment. The UKMCR has recorded prospective data from patients prescribed CBMPs by Curaleaf Clinic since December 2019 with ethical approval from the Central Bristol Ethics Committee (22/SW/0145) [9]. Written and informed consent was provided consecutively by all patients. Medications were initiated in line with UK regulations. The diagnosis of endometriosis and confirmation of previous treatment with licensed medications to address endometriosis‐associated pain was identified from primary care records. Data on the time frame from index surgery to treatment were not recorded.

Patients with incomplete baseline PrOMs, enrolled for less than 18 months, or without a primary indication of endometriosis were excluded from the analysis. Data were extracted on 13th December 2023.

### 2.2 Data Collection

Clinicians collected demographic data at initial assessment. The indication(s) for treatment and relevant co‐morbidities were recorded. The Charlson Comorbidity Index was calculated for each patient [10]. Data on tobacco, alcohol and cannabis use were collected. Lifetime cannabis use was calculated in gram years. CBMP treatment details were recorded throughout. Changes to other medications were also reported by patients or clinicians. Daily prescribed opiate doses were calculated in oral morphine equivalents (mg/day) [11].

#### 2.2.1 Patient‐Reported Outcome Measures

Pain‐specific and HRQoL PrOMs were collected via a bespoke online web‐based platform at baseline and 1, 3, 6, 12 and 18 months intervals.

The Short‐Form McGill Pain Questionnaire‐2 (McGill) measures the characteristics and severity of pain. Questions are organised into subscales: continuous, intermittent, neuropathic and affective pain [12]. Each of these is ranked from 0 (none) to 10 (worst possible) [13].

The Brief Pain Inventory Short Form (BPI) measures pain severity and interference. Pain severity and interference are both calculated on a 10‐point scale. Pain severity ranges from 0 (no pain) to 10 (pain as awful as you can imagine) and pain interference ranges from 0 (no interference) to 10 (complete interference) [14].

Pain Visual Analogue Scale (VAS) uses a horizontal 10 cm line, anchored to 0 (no pain) and 10 (worst possible pain) [15]. The patients are asked to rate their pain severity over the last 24 h.

The generalised anxiety disorder‐7 (GAD‐7) is used to screen and assess the severity of generalised anxiety disorder (GAD) between 0 and 21 [16]. GAD severity is classified into mild, moderate and severe anxiety by scores of ≥ 5, ≥ 10 and ≥ 15, respectively [16].

The single‐item sleep quality scale (SQS) is a self‐reported assessment of sleep quality. The patient ranks their sleep using a numerical value between 0 and 10, corresponding to terrible (0), poor [1, 2, 3], fair [4, 5, 6], good [7, 8, 9] and excellent [10] sleep quality over 7 days [17].

EQ‐5D‐5L assesses HRQoL across 5 domains: ‘mobility’, ‘self‐care’, ‘usual activities’, ‘pain/discomfort’ and ‘anxiety/depression’ on a scale from 1 to 5 (‘1’ = ‘no problems’ to ‘5’ = ‘extreme problems’) [18]. A 5‐digit health state is subsequently mapped to a UK‐specific index value [19]. The maximum value for the index value is 1 [19].

Patient global impression of change score (PGIC) assesses the patient's perception of improvement in symptoms since commencing the treatment. The patient is asked to rate their improvements in activity limitations, symptoms, emotions and overall quality of life on a 7‐point scale (1 = ‘no change’ to 7 = ‘a great deal better’) [20].

#### 2.2.2 Adverse Events

AEs were either self‐reported at each follow‐up interval or at the time of the event virtually. Additionally, AEs could be recorded during routine follow‐up appointments with clinicians if they were unrecorded online. AEs were recorded in accordance with the common terminology for adverse events version 4.0 [21].

#### 2.2.3 Missing Data

If PrOM data was missing during the follow‐up period, it was handled using the baseline observation carried forward (BOCF) approach [22].

### 2.3 Statistical Analysis

Descriptive statistics were performed on patient demographics, drug, alcohol and cannabis data and reported AEs. Parametric data were presented as mean ± standard deviation (SD) and nonparametric data were presented as median and interquartile range (IQR).

A repeated measures one‐way ANOVA was used to compare changes in PrOMs at each timepoint. For PrOMs with a significant finding on repeated measures ANOVA, post hoc pairwise comparison with a Bonferroni correction was conducted to identify which timepoints significantly differ from each other while controlling for multiple comparisons.

To determine the clinical significance improvements for patients the minimal clinically important difference (MCID), moderately important improvements (MII) and substantially important improvements (SII) were calculated by detecting a reduction of 1 point, ≥ 30% and ≥ 50% respectively [23].

Statistical significance was defined as *p* < 0.050. Statistical analysis was performed using Statistical Package for Social Sciences [IBM Statistics version 29 SPSS Inc. (New York, IL), USA].

## 3 Results

Data was extracted on December 13, 2023, with 19 763 patients enrolled in the UKMCR. After exclusion criteria were applied, 63 patients were included in the final analysis. Patients were excluded sequentially, for not completing a baseline PrOM (*n* = 1105, 8.77%), being enrolled with the UKMCR for < 18 months (*n* = 13 684, 69.24%) and for a primary diagnosis that was not endometriosis (*n* = 4911, 24.85%).

### 3.1 Demographics

The mean age and body mass index were 33.71 ± 6.48 years and 26.07 ± 5.81 kg/m^2^ respectively (Table 1). Over one‐third (*n* = 24, 38.10%) were current cannabis users at baseline, with median lifetime use of 4.00 [1.00–9.75] gram years. *For specific comorbidities see* Table S1.

**TABLE 1:** Demographic details and smoking, alcohol and cannabis history at baseline assessment. Data for patients prescribed cannabis‐based medicinal products for a primary indication of endometriosis was recorded by clinicians.

Demographic details	*n* (%)/mean ± SD
Gender	
Female	63 (100%)
Age	33.71 ± 6.48
Body mass index, kg/m^2^	26.07 ± 5.81
Occupation	
Clerical support workers	5 (7.94%)
Craft and related trades workers	3 (4.76%)
Elementary occupations	2 (3.17%)
Managers	3 (4.76%)
Other occupations	6 (9.52%)
Plant and machine operators and assemblers	0 (0.00%)
Professional	15 (23.81%)
Service and sales workers	5 (7.94%)
Skilled agricultural, forestry and fishery workers	0 (0.00%)
Technicians and associate professionals	4 (6.35%)
Undisclosed	0 (0.00%)
Unemployed	20 (31.75%)
Charlson comorbidity score	0 [0.00–0.00]

Smoking, alcohol and cannabis status	*n* (%)/median [IQR]
Smoking status	
Ex‐smoker	25 (39.68%)
Current smoker	11 (17.46%)
Never smoked	27 (42.86%)
Smoking pack years	6.50 [2.00–15.00]
Weekly alcohol consumption, units	0.00 [0.00–2.00]
Cannabis status	
Ex‐user	16 (25.40%)
Current user	24 (38.10%)
Cannabis naïve	23 (36.51%)
Lifetime cannabis use, gram years	4.00 [1.00–9.75]

*Note:* Data on patient smoking, alcohol and cannabis history was collected by clinicians. ‘Never smoked’ patients had never used cannabis prior to their prescription, ‘Ex‐smoker’ patients had previously used cannabis but were not currently using alongside their prescriptions, and ‘Current smoker’ patients were using other cannabis alongside their prescription cannabis. Data is presented as either *n* (%), mean ± SD, or median [IQR].

Abbreviations: IQR, interquartile range; *n*, number of patients (63); SD, standard deviation.

### 3.2 Cannabis‐Based Medicinal Products

CBMP dosing is displayed in Table S2. The median CBD dose was 20.00 [20.00–21.00] mg/24 h and 30.00 [20.00–55.00] mg/24 h at baseline and 18 months respectively. The median Δ9‐THC dose was 18.72 [1.00–21.00] mg/24 h and 110.00 [85.00–215.00] mg/24 h at baseline and 18 months respectively. Most patients at 18 months (39, 61.90%) were prescribed both oils and dried flowers. Of the remaining patients, 11 (17.46%) were prescribed dried flowers only and 13 (20.63%) were prescribed oils only.

### 3.3 Patient‐Reported Outcome Measures

The rate of non‐completion of PrOMs at baseline, 1, 3, 6, 12 and 18 months was 1.6%, 7.9%, 33.3%, 55.6% and 69.8% respectively.

Changes were observed in the Pain VAS (*p* = 0.001), BPI interference (*p* < 0.001) and severity (*p* < 0.001) and McGill intermittent (*p* < 0.001), continuous (*p* < 0.001), neuropathic (*p* = 0.019), affective (*p* < 0.001) and total (*p* < 0.001) scales (Table 2). Differences were also determined for EQ‐5D‐5L index (*p* < 0.001), self‐care (*p* = 0.029), usual activities (*p* < 0.001), pain and discomfort (*p* < 0.001) and depression and anxiety (*p* < 0.001) subscales. No improvement was determined for the EQ‐5D‐5L mobility score (*p* = 0.186). Finally, changes were also recorded in the GAD‐7 (*p* = 0.001), SQS (*p* = 0.001) and PGIC (*p* = 0.027).

**TABLE 2:** Repeated one‐way ANOVA showing statistical differences in pain‐specific and HRQoL PrOMS.

PrOM	Baseline mean ± SD	1 month mean ± SD	3 months mean ± SD	6 months mean ± SD	12 months mean ± SD	18 months mean ± SD	*F*	*p*
PAIN VAS	6.94 ± 2.24	5.83 ± 2.56	5.76 ± 2.48	6.06 ± 2.61	6.41 ± 2.39	6.57 ± 2.33	4.87	0.001
McGill total	5.03 ± 1.78	3.83 ± 2.04	3.77 ± 1.90	4.01 ± 2.04	3.99 ± 1.88	3.96 ± 1.82	11.686	< 0.001
McGill affective	5.85 ± 2.53	4.19 ± 2.56	4.22 ± 2.70	4.92 ± 2.79	5.09 ± 2.80	5.40 ± 2.82	11.375	< 0.001
McGill continuous	6.30 ± 1.91	4.98 ± 2.42	5.29 ± 2.37	5.52 ± 2.58	5.92 ± 2.26	5.80 ± 2.26	6.389	< 0.001
McGill intermittent	5.26 ± 2.19	4.00 ± 2.38	3.85 ± 2.32	4.32 ± 2.32	4.68 ± 2.49	4.86 ± 2.38	8.640	< 0.001
BPI interference	6.46 ± 2.41	5.14 ± 2.69	5.28 ± 2.49	5.69 ± 2.73	5.66 ± 2.63	5.92 ± 2.70	5.914	< 0.001
BPI Severity	5.62 ± 1.61	4.58 ± 1.84	4.67 ± 1.71	5.07 ± 1.94	5.07 ± 1.68	5.12 ± 1.71	6.641	< 0.001
EQ‐5D‐5L index	0.37 ± 0.31	0.59 ± 0.26	0.55 ± 0.26	0.50 ± 0.30	0.47 ± 0.29	0.46 ± 0.30	12.153	< 0.001
EQ‐5D‐5L mobility	2.38 ± 1.08	2.14 ± 1.09	2.13 ± 1.02	2.21 ± 1.08	2.25 ± 1.02	2.29 ± 1.05	1.56	0.186
EQ‐5D‐5L selfcare	1.83 ± 1.04	1.59 ± 0.94	1.56 ± 0.82	1.79 ± 1.02	1.73 ± 0.94	1.67 ± 0.90	2.795	0.029
EQ‐5D‐5L usual activities	2.78 ± 1.26	2.22 ± 1.17	2.30 ± 1.15	2.43 ± 1.15	2.56 ± 1.06	2.52 ± 1.18	5.226	< 0.001
EQ‐5D‐5L pain and discomfort	3.63 ± 0.99	2.73 ± 0.97	2.89 ± 0.90	3.08 ± 1.02	3.25 ± 0.99	3.27 ± 1.05	12.682	< 0.001
EQ‐5D‐5L depression and anxiety	2.57 ± 1.10	2.13 ± 0.91	2.14 ± 0.99	2.24 ± 0.99	2.37 ± 1.11	2.41 ± 1.07	5.535	< 0.001
GAD‐7	7.56 ± 5.90	5.30 ± 4.72	5.79 ± 5.53	5.98 ± 5.16	6.65 ± 5.46	6.43 ± 5.37	5.75	0.001
SQS	4.97 ± 2.55	6.35 ± 2.46	5.94 ± 2.52	5.14 ± 2.52	5.90 ± 2.55	5.46 ± 2.55	4.92	0.001
PGIC	n/a ± n/a	5.21 ± 1.27	5.62 ± 0.88	5.53 ± 1.16	5.59 ± 1.09	5.43 ± 1.29	3.152	0.027

*Note:* Repeated one‐way ANOVA for Pain VAS = Pain Visual Analogue Scale, Short‐Form McGill Pain Questionnaire‐2 (McGill) pain score, Brief Pain Inventory (BPI) Interference and Severity scores, EQ‐5D‐5L = EuroQol‐5 Dimension, GAD‐7 = generalised anxiety disorder‐7, SQS = single‐item sleep quality scale, PGIC = patient global impression of change. A repeated one‐way ANOVA was used to identify improvements in PrOMs over 18 months of treatment with cannabis‐based medicinal products. For BPI, McGill and GAD‐7, a lower score indicates an improvement. For all other PrOMs, a greater score indicates an improved outcome. Data is presented as mean ± SD.

**FIGURE 1:**
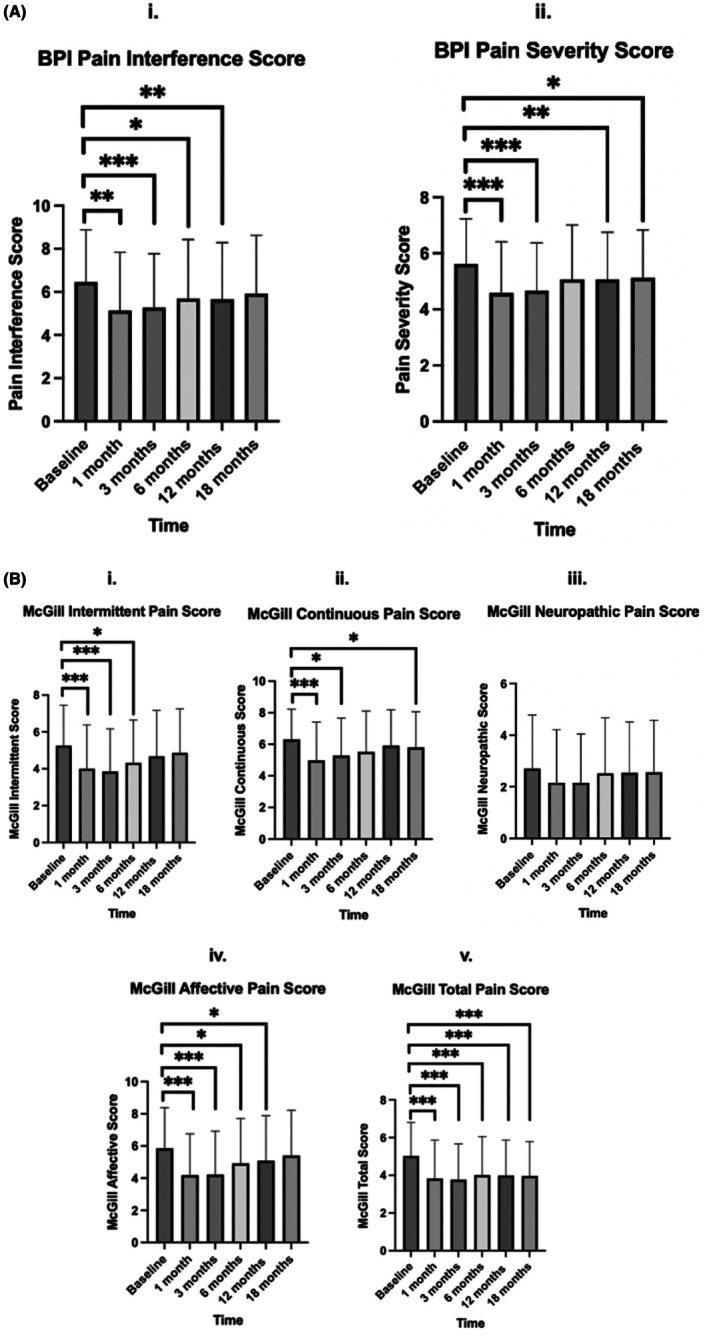
Paired baseline and follow‐up scores for Brief Pain Inventory (BPI) (A) and Short‐Form McGill Pain Questionnaire‐2 (McGill) (B). Graphs were only presented for pairwise comparisons compared to baseline for clarity. All comparisons can be found in Table S2. The level of significance compared to baseline is presented through asterisks. If the difference was not significant it was left blank for clarity, however all *p*‐values can be found in Table S2. Scores presented as mean ± SD. *p* < 0.001 = ***, *p* < 0.010 = **, *p* < 0.005 = *.

Pairwise comparisons for all PrOMS that were significant on repeated measures ANOVA are detailed in Tables S3–S16. Figure 1 displays the post hoc tests of pain‐specific PrOMs comparing baseline to each follow‐up. The BPI interference subscale (Figure 1Ai) showed improvements between baseline and 1, 3 and 12 months follow‐ups (*p* > 0.050), but not at 18 months. The BPI severity subscale (Figure 1Aii) showed improvements between baseline and 1, 3, 12 and 18 months follow‐ups (*p* < 0.050), but not at 6‐months. The McGill intermittent subscale (Figure 1Bi) showed significant improvement between baseline and 1‐, 3‐ and 6‐month follow‐ups (*p* < 0.050), but not at 12‐ or 18‐months. The continuous subscale (Figure 1Bii) demonstrated significant improvement from baseline at 1‐, 3‐ and 18‐month follow‐ups (*p* < 0.050), but not at 6‐, or 12‐months. The affective subscale (Figure 1Biv) showed an improvement between baseline and 1‐, 3‐, 6‐ and 12‐month follow‐ups (*p* < 0.050), but not at 18‐months. However, the McGill neuropathic score (Figure 1Biii) showed no improvements at any time point compared to baseline (*p* > 0.050). The McGill total score (Figure 1Bv) showed an improvement between baseline and all follow‐ups (*p* < 0.001).

The proportion of individuals reporting MCID, MII and SII for pain outcomes at each time point is detailed in Tables S17–S19.

There were improvements in the GAD‐7 from baseline to 1‐ and 3‐months (*p* < 0.050). There was also an improvement in the SQS from baseline to 1 and 12 months. However, the score was lower at 6‐months compared to 1‐month (*p* = 0.033). Pain VAS showed a significant difference between baseline, and 1‐, 3‐ and 6‐months (*p* < 0.005).

EQ‐5D‐5L usual activities and depression and anxiety subscales displayed increases between baseline, and 1‐ and 3‐month (*p* < 0.050). EQ‐5D‐5L index value and the pain and discomfort subscale were improved from baseline to 1‐, 3‐, 6‐, 12‐ and 18‐month. The scores at 12‐ and 18‐month for the EQ‐5D‐5L index value were lower than at 1‐month (*p* < 0.050). All other pairwise comparisons were not statistically significant (*p* > 0.050).

### 3.4 Adverse Effects

A total of 62 AEs were reported by 16 (25.40%) patients (Table 3). Eighty‐one (50%) were mild, 69 (42.59%) were moderate, 11 (6.79%) were severe and 1 (0.62%) was life threatening/disabling. The AE with the highest incidence was fatigue (*n* = 16, 9.66%) and the disabling AE was a urinary tract infection (0.62%).

**TABLE 3:** Adverse event incidence and severity level after 18 months of CBMP use.

Adverse event	Mild	Moderate	Severe	Life threatening/disabling	Total (*n*, %)
Abdominal pain	7	2	0	0	9 (14.29%)
Amnesia	0	1	0	0	1 (1.59%)
Anorexia	0	2	0	0	2 (3.17%)
Arthralgia	0	1	0	0	1 (1.59%)
Concentration impairment	4	1	0	0	5 (7.94%)
Confusion	3	1	0	0	4 (6.35%)
Constipation	10	0	0	0	10 (15.87%)
Delirium	3	0	0	0	3 (4.76%)
Dizziness	4	2	0	0	6 (9.52%)
Dry mouth	9	3	0	0	12 (19.05%)
Dyspepsia	4	2	0	0	6 (9.52%)
Fall	1	0	0	0	1 (1.59%)
Fatigue	3	11	2	0	16 (25.40%)
Generalised muscle weakness	1	1	2	0	4 (6.35%)
Headache	6	6	1	0	13 (20.63%)
Insomnia	4	4	4	0	12 (19.05%)
Lethargy	8	7	0	0	15 (23.81%)
Lower gastrointestinal haemorrhage	1	0	0	0	1 (1.59%)
Lung infection	0	5	0	0	5 (7.94%)
Nausea	6	0	0	0	6 (9.52%)
Pharyngitis	0	4	1	0	5 (7.94%)
Rash NOS	2	0	0	0	2 (3.17%)
Somnolence	0	10	1	0	11 (17.46%)
Spasticity	2	0	0	0	2 (3.17%)
Urinary tract infection	0	4	0	1	5 (7.94%)
Vertigo	0	1	0	0	1 (1.59%)
Vomiting	1	0	0	0	1 (1.59%)
Weight loss	3	0	0	0	3 (4.76%)
Total (*n*, %)	81 (128.57%)	69 (109.52%)	11 (17.46%)	1 (1.59%)	162 (257.14%)

*Note:* Adverse events were categorised as mild, moderate, severe or life‐threatening/disabling. Adverse events were calculated by dividing the total number of adverse events by the number of patients experiencing adverse events. A percentage of each adverse event as well as the total percentage of all adverse events was presented. *n* = 162.

### 3.5 Opioid Medications

Thirty (47.62%) patients were prescribed opioids during the studied period. The mean oral morphine equivalent at baseline was 21.20 ± 15.05 mg/day. There was a difference across analysis on repeated measures ANOVA (*p* = 0.015). However, after application of Bonferroni correction there was no difference between the baseline value and 1 (22.53 ± 19.39 mg/day; *p* = 1.000), 3 (20.93 ± 20.20 mg/day; *p* = 1.000), 6 (15.93 ± 15.28 mg/day; *p* = 0.347), 12 (15.93 ± 15.28 mg/day; *p* = 0.347) and 18 months (14.60 ± 14.84; *p* = 0.071).

## 4 Discussion

This observational study used data from the UKMCR to assess changes in endometriosis patients treated with CBMPs. It indicates that treatment with CBMPs is associated with improvements in all pain‐specific PrOMs, with the McGill Pain Score, BPI Interference (exc. 18–6 months), BPI Severity (exc. 12 months) and Pain VAS (exc. 18 months) scores in patients with endometriosis‐associated chronic pain. Improvements were also seen in general HRQoL, largely secondary to improvements in the Pain and Discomfort domain. As highlighted, these changes were not always present at long‐term follow‐up which may relate to pharmacological tolerance or the methods used to address attrition bias. CBMPs were well tolerated, with most patients (*n* = 47, 74.30%) reporting no adverse events or 16 (25.70%) patients experiencing mostly mild to moderate adverse events, and 1 (1.59%) life‐threatening adverse event (urinary tract infection).

Treatment was associated with improvements in McGill total value (*p* < 0.001) at all periods following baseline. Notably, the affective pain subscale score improved at all timepoints except at 18 months post‐baseline, indicating a reduction in the emotional impact of pain over time, consistent with the overall improvement of HRQoL PrOMs. Variations might occur compared to previous analyses from the UKMCR which encompass a range of pain conditions beyond endometriosis, impeding direct comparison [24]. Heterogeneity in CBMPs and prior cannabis use complicates the generalisation of findings between studies. Additionally, sex‐specific responses in chronic pain to CBMPs may be reflected in the present study [25].

Furthermore, pain VAS scores showed improvement at 1, 3 and 6 months following baseline, consistent with findings from a previous UKMCR study [7]. At 18 months, approximately 20% of individuals reported a clinically significant change in their pain severity or interference. When excluding those patients with complete data up to 18 months, over 60% of participants reported a MCID. A meta‐analysis of RCTs modelled that 10% of chronic pain patients experience a clinically significant improvement in pain in response to CBMPs [8]. The reasons for differences could be multifactorial. The meta‐analysis only included one RCT of endometriosis‐associated pain. Moreover, the meta‐analysis did not include studies that assessed inhaled preparations of CBMPs. Almost 80% of participants were prescribed dried flower in isolation or in combination with oil at 18 months in the present study.

Across pain‐specific PrOMs, improvements were greater at earlier follow‐ups. This trend may be due to increasing rates of incomplete PrOMs at later time points. Use of the BOCF method will have biased the outcome towards a null finding. Moreover, an exaggerated placebo response to CBMPs may have influenced this trend [26]. Finally, this trajectory could represent pharmacological tolerance [27]. Further studies are necessary to evaluate this further.

The study has limitations that should be considered. The sample size was small, limiting the reliability of the study findings and preventing sub‐group analysis. The observational design of this study also means a cause‐and‐effect relationship cannot be determined. The results reported may be secondary to the initiation of other therapies or surgical excision during the studied period, which was not recorded. Furthermore, CBMPs have been shown to provide an exaggerated placebo effect due to their distinct aroma and capacity to induce vasoactive and psychoactive effects [28]. Expectancy bias is high among individuals receiving CBMPs, driven by positive media coverage and the hype surrounding CBMPs [26]. These could increase the likelihood of a false positive. To compare to recent RCT data, 30.9% and 23.5% reported a clinically significant improvement in non‐menstrual pelvic pain and dysmenorrhoea to placebo in the EDELWEISS 3 trial at 3 months. In the present study 44.44% and 47.62% reported a MCID in the BPI Interference and Severity subscales respectively at 3 months [29].

The study is subject to selection bias. Almost 2 in 5 participants were cannabis consumers at baseline. This may contribute to the development of pharmacological tolerance. Conversely, these may be self‐identified responders to CBMP therapy and therefore more likely to report a positive effect. Furthermore, the high rate of concurrent opioid prescriptions (47.62%) may suggest that this cohort represents women at the more severe end of the endometriosis pain‐related spectrum. In addition, treatment was conducted at a private clinic, where the ability to enroll is influenced by financial considerations. Moreover, the need to pay for treatment may also increase expectancy of the effects of CBMPs.

This study experienced attrition bias, with a PrOMs incompletion rate of 69.8% at 18 months. Although reasons for dropout were not collected, potential factors may include adverse events, lack of efficacy, family planning, cost of treatment or change of treatment provider. While the use of the BOCF method is conservative, the dropout rate prevents a definitive conclusion regarding whether the observed benefits are solely attributable to CBMP treatment.

RCTs are required to address the paucity of high‐quality evidence and the limitations of the present study. These should aim to recruit cannabis‐naïve patients or utilise a satisfactory washout period to reduce the confounding effect of prior cannabis use. The high prevalence of CBMP flower prescribing in this study, suggests these may be the most appropriate products to compare against placebo or standard of care. However, considering the challenges with precise standardisation of active pharmaceutical ingredients in dried flower products across batches and the dose delivered by medical vapourisers, oils may be a more pragmatic starting point for building evidence for this class of medications.

Overall, these results provide a signal towards improvement in short‐term pain severity and interference for endometriosis patients after the initiation of CMBP treatment. However, due to the design of this study, a causal effect cannot be determined. Moreover, analysis of the long‐term effects and whether patients develop pharmacological tolerance is necessary. This study provides valuable real‐world data and complements the development of RCTs to further examine the efficacy and safety of CBMPs for endometriosis‐associated chronic pain.

Overall, these results provide a signal towards improvement in short‐term pain severity and interference for endometriosis patients after the initiation of CMBP treatment, although there was diversity at different pain intervals. However, due to the design of this study, a causal effect cannot be determined and these results demonstrate a need for RCTs to examine the effect of CMBP treatment on endometriosis‐associated chronic pain. Large loss to follow up and the lack of a comparator mean the results from this study are unable to justify changes to clinical practice at this stage.

## Funding

The authors have nothing to report.

## Conflicts of Interest

Sara Getter was a biomedicine student and current MPH student at Imperial College London. Sara Getter has no shareholdings in pharmaceutical companies. Simon Erridge is a resident doctor and Research Director at Curaleaf Clinic. Simon Erridge is a research fellow at Imperial College London. Simon Erridge has no shareholdings in pharmaceutical companies. John Warner‐Levy is a medical student at Imperial College London. John Warner‐Levy has no shareholdings in pharmaceutical companies. Evonne Clarke is the Patient Care Director at Curaleaf Clinic. Evonne Clarke has no shareholdings in pharmaceutical companies. Katy McLachlan is the Chief Pharmacist at Curaleaf Clinic. Katy McLachlan has no shareholdings in pharmaceutical companies. Ross Coomber is a consultant orthopaedic surgeon at St George's Hospital, London and Operations Director at Curaleaf Clinic. Ross Coomber has no shareholdings in pharmaceutical companies. Shelley Barnes is a consultant pain specialist at North Bristol NHS Trust and Curaleaf Clinic. Shelley Barnes has no shareholdings in pharmaceutical companies. Alia Darweish Medniuk is a consultant pain specialist at North Bristol NHS Trust and Curaleaf Clinic. Alia Darweish Medniuk has no shareholdings in pharmaceutical companies. Rahul Guru is a consultant pain specialist at Cardiff and Vale University Health Board and Curaleaf Clinic. Rahul Guru has no shareholdings in pharmaceutical companies. Wendy Holden is a consultant pain specialist at Curaleaf Clinic. Wendy Holden has no shareholdings in pharmaceutical companies. Mohammed Sajad is a consultant pain specialist at Curaleaf Clinic. Mohammed Sajad has no shareholdings in pharmaceutical companies. Robert Searle is a consultant pain specialist at Curaleaf Clinic. Robert Searle has no shareholdings in pharmaceutical companies. Azfer Usmani is a consultant pain specialist at Curaleaf Clinic. Azfer Usmani has no shareholdings in pharmaceutical companies. Sanjay Varma is a consultant pain specialist at Curaleaf Clinic. Sanjay Varma has no shareholdings in pharmaceutical companies. James J. Rucker is a consultant psychiatrist at Curaleaf Clinic. James Rucker is an honorary consultant psychiatrist at The South London & Maudsley NHS Foundation Trust and an NIHR Clinician Scientist Fellow at the Centre for Affective Disorders at King's College London. James Rucker is funded by a fellowship (CS‐2017‐17‐007) from the National Institute for Health Research (NIHR). The views expressed are those of the author(s) and not necessarily those of the NHS, the NIHR or the Department of Health. James Rucker leads the Psychedelic Trials Group at King's College London. King's College London receives grant funding from COMPASS Pathways PLC to undertake phase 1 and phase 2 trials with psilocybin. COMPASS Pathways PLC has paid for James Rucker to attend trial‐related meetings and conferences to present the results of research using psilocybin. James Rucker has undertaken paid consultancy work for Beckley PsyTech and Clerkenwell Health. Payments for consultancy work are received and managed by King's College London and James Rucker does not benefit personally. James Rucker has no shareholdings in pharmaceutical companies. Michael Platt is a consultant in pain services at Curaleaf Clinic. Michael Platt has no shareholdings in pharmaceutical companies. Mikael H. Sodergren is a consultant hepatopancreatobiliary surgeon at Imperial College NHS Trust, London, a senior clinical lecturer at Imperial College London and the chief medical officer of Curaleaf International. Mikael Sodergren has no shareholdings in pharmaceutical companies.

## Supporting information


**Data S1:** ajo70078‐sup‐0001‐DataS1.docx.
