# Supplementary material for: A Longitudinal Assessment of Endometriosis Patients Prescribed Cannabis‐Based Medicinal Products: A Case Series From the UK Medical Cannabis Registry
Source: Aust N Z J Obstet Gynaecol. 2025 Nov 26;66(1):e70078. doi: 10.1111/ajo.70078 (PMC12920047; doi:10.1111/ajo.70078)
Supplement: Supplementary file 1 — Data S1: ajo70078‐sup‐0001‐DataS1.docx. [file AJO-66-0-s001.docx]

***Supplementary Table 1. Comorbidities at baseline assessment.***

*Comorbidities for patients prescribed cannabis-based medicinal products for a primary indication of endometriosis was recorded by clinicians. SD – standard deviation. Data is presented as either n (%), mean ± SD. n – number of patients (63)*

| **Comorbidities** | | **n (%)** |
| --- | --- | --- |
|  | Anxiety/depression | 26 (41.27%) |
|  | Arthritis | 4 (6.35%) |
|  | Cerebrovascular accident or transient ischemic attack | 0 (0.00%) |
|  | Chronic obstructive pulmonary disease | 1 (1.59%) |
|  | Congestive heart failure | 0 (0.00%) |
|  | Connective tissue disease | 4 (6.35%) |
|  | Dementia | 0 (0.00) |
|  | Diabetes |  |
|  | End organ damage | 1 (1.59%) |
|  | Uncomplicated | 1 (1.59%) |
|  | None or diet – controlled | 61 (96.83%) |
|  | Endocrine thyroid dysfunction | 2 (3.17%) |
|  | Epilepsy | 0 (0.00%) |
|  | Hemiplegia | 0 (0.00%) |
|  | Hypertension | 0 (0.00%) |
|  | Liver disease | 0 (0.00%) |
|  | Moderate to severe chronic kidney disease | 0 (0.00%) |
|  | Myocardial infarction | 0 (0.00%) |
|  | Peptic ulcer disease | 1 (1.59%) |
|  | Peripheral vascular disease | 0 (0.00%) |
|  | Solid tumor | 1 (1.59%) |
|  | VTE | 0 (0.00%) |

***Supplementary Table 2. Prescribed Cannabis-Based Medicinal Products***

*Patients were treated with either oils, dried flower, or a combination of both, displayed in the table as n, %. The dose of cannabidiol (CBD) and (−)-trans-Δ9-tetrahydrocannabinol (Δ9-THC) is displayed as mg/24 and presented as median [IQR]. IQR = interquartile range. n – number of patients (63).*

|  | n (%)/ median [IQR] | | | | | |
| --- | --- | --- | --- | --- | --- | --- |
|  | **Baseline** | **1-month** | **3-months** | **6-months** | **12-months** | **18-months** |
| **Administration** |  |  |  |  |  |  |
| Oils | 24 (38.10%) | 21  (33.33%) | 18  (28.57%) | 12  (19.05%) | 12  (19.05%) | 13  (20.63%) |
| Dry Flower | 5  (7.94%) | 4  (6.35%) | 3  (4.76%) | 6  (9.52%) | 11  (17.46%) | 11  (17.46%) |
| Oils and Dry Flower | 34  (53.97%) | 38  (60.32%) | 42  (66.67%) | 45  (71.43%) | 40  (64.49%) | 39  (61.90%) |
| **Dosage (milligrams/day)** |  |  |  |  |  |  |
| CBD | 20.00  [20.00-21.00] | 20.00  [20.00-25.00] | 20.00  [20.00-30.00] | 25.00  [20.00-30.00] | 25.00  [15.00-54.00] | 30.00  [20.00-55.00] |
| THC | 18.72 [1.00-21.00] | 100.00  [10.00-110.00] | 102.50  [13.00-110.72] | 105.00  [85.72-120.00] | 110.00  [85.00-205.00] | 110.00  [85.00-215.00] |

***Supplementary Table 3. Post-hoc pairwise comparison analysis of Brief Pain Inventory Short Form Severity Subscale.***

*Data is presented as mean difference ± standard deviation (SD) with the later follow up subtracted from the earlier follow up*

| **Follow-up Interval** | **Mean ± SD, *p-*value** | | | | | |
| --- | --- | --- | --- | --- | --- | --- |
|  | **Baseline** | **1-month** | **3-months** | **6-months** | **12-months** | **18-months** |
| Baseline |  |  |  |  |  |  |
| 1 | 1.04 ± 1.72 p < 0.001 |  |  |  |  |  |
| 3 | 0.94 ± 1.64 p < 0.001 | -0.10 ± 1.81 p = 1.000 |  |  |  |  |
| 6 | 0.55 ± 1.54 p = 0.097 | -0.49 ± 1.90 p = 0.675 | -0.40 ± 1.75 p = 1.000 |  |  |  |
| 12 | 0.55 ± 1.14 p = 0.004 | -0.49 ± 1.83 p = 0.574 | -0.39 ± 1.74 p = 1.000 | 0.00 ± 1.33 p = 1.000 |  |  |
| 18 | 0.50 ± 1.10 p = 0.011 | -0.54 ± 1.87 p = 0.362 | -0.45 ± 1.78 p = 1.000 | -0.05 ± 1.60 p = 1.000 | -0.06 ± 1.13 p = 1.000 |  |

***Supplementary Table 4. Post-hoc pairwise comparison analysis of Brief Pain Inventory Short Form Interference Subscale.***

*Data is presented as mean difference ± standard deviation (SD) with the later follow up subtracted from the earlier follow up*

| **Follow-up Interval** | **Mean ± SD, *p-*value** | | | | | |
| --- | --- | --- | --- | --- | --- | --- |
|  | **Baseline** | **1-month** | **3-months** | **6-months** | **12-months** | **18-months** |
| Baseline |  |  |  |  |  |  |
| 1 | 1.32 ± 2.65 p = 0.003 |  |  |  |  |  |
| 3 | 1.18 ± 2.09 p < 0.001 | -0.14 ± 2.32 p = 1.000 |  |  |  |  |
| 6 | 0.77 ± 1.98 p = 0.045 | -0.55 ± 2.40 p = 1.000 | -0.41 ± 1.93 p = 1.000 |  |  |  |
| 12 | 0.80 ± 1.76 p = 0.009 | -0.52 ± 2.90 p = 1.000 | -0.37 ± 2.30 p = 1.000 | -0.03 ± 2.00 p = 1.000 |  |  |
| 18 | 0.54 ± 1.41 p = 0.054 | -0.78 ± 2.64 p = 0.341 | -0.64 ± 2.31, p = 0.488 | -0.23 ± 1.63 p = 1.000 | -0.26 ± 1.71 p = 1.000 |  |

***Supplementary Table 5. Post-hoc pairwise comparison analysis of Short-Form McGill Pain Questionnaire-2 Total Score.***

*Data is presented as mean difference ± standard deviation (SD) with the later follow up subtracted from the earlier follow up*

| **Follow-up Interval** | **Mean ± SD, *p-*value** | | | | | |
| --- | --- | --- | --- | --- | --- | --- |
|  | **Baseline** | **1-month** | **3-months** | **6-months** | **12-months** | **18-months** |
| Baseline |  |  |  |  |  |  |
| 1 | 1.19 ± 1.75 p < 0.001 |  |  |  |  |  |
| 3 | 1.26 ± 1.80 p < 0.001 | 0.06 ± 1.57 p = 1.000 |  |  |  |  |
| 6 | 1.02 ± 1.82 p = 0.045 | -0.18 ± 1.69 p = 1.000 | -0.24 ± 1.22 p = 1.000 |  |  |  |
| 12 | 1.04 ± 1.87 p = 0.009 | -0.16 ± 1.69 p = 1.000 | -0.22 ± 1.10 p = 1.000 | 0.02 ± 1.14 p = 1.000 |  |  |
| 18 | 1.07 ± 1.79 p = 0.054 | -0.13 ± 1.71 p = 1.000 | -0.19 ± 1.17 p = 0.488 | 0.05 ± 1.26 p = 1.000 | 0.03 ± 0.78 p = 1.000 |  |

***Supplementary Table 6. Post-hoc pairwise comparison analysis of Short-Form McGill Pain Questionnaire-2 Affective Subscale***

*Data is presented as mean difference ± standard deviation (SD) with the later follow up subtracted from the earlier follow up*

| **Follow-up Interval** | **Mean ± SD, *p-*value** | | | | | |
| --- | --- | --- | --- | --- | --- | --- |
|  | **Baseline** | **1-month** | **3-months** | **6-months** | **12-months** | **18-months** |
| Baseline |  |  |  |  |  |  |
| 1 | 1.67 ± 2.23 p < 0.001 |  |  |  |  |  |
| 3 | 1.63 ± 2.37 p < 0.001 | -0.04 ± 2.06 p = 1.000 |  |  |  |  |
| 6 | 0.93 ± 2.25 p = 0.024 | -0.74 ± 2.18 p = 0.140 | -0.70 ± 1.91 p = 0.077 |  |  |  |
| 12 | 0.77 ± 1.71 p = 0.010 | -0.90 ± 2.64 p = 0.128 | -0.87 ± 2.46 p = 0.104 | -0.17 ± 2.21 p = 1.000 |  |  |
| 18 | 0.45 ± 1.34 p = 0.154 | -1.22 ± 2.57 p = 0.006 | -1.18 ± 2.47 p = 0.005 | -0.48 ± 2.31 p = 1.000 | -0.32 ± 1.67 p = 1.000 |  |

***Supplementary Table 7. Post-hoc pairwise comparison analysis of Short-Form McGill Pain Questionnaire-2 Continuous Subscale***

*Data is presented as mean difference ± standard deviation (SD) with the later follow up subtracted from the earlier follow up*

| **Follow-up Interval** | **Mean ± SD, *p-*value** | | | | | |
| --- | --- | --- | --- | --- | --- | --- |
|  | **Baseline** | **1-month** | **3-months** | **6-months** | **12-months** | **18-months** |
| Baseline |  |  |  |  |  |  |
| 1 | 1.32 ± 2.36 p < 0.001 |  |  |  |  |  |
| 3 | 1.02 ± 2.09 p = 0.004 | -0.30 ± 2.40 p = 1.000 |  |  |  |  |
| 6 | 0.78 ± 2.25 p = 0.114 | -0.54 ± 2.47 p = 0.140 | -0.24 ± 1.94 p = 1.000 |  |  |  |
| 12 | 0.39 ± 1.52 p = 0.735 | -0.93 ± 2.56 p = 0.076 | -0.63 ± 2.10 p = 0.301 | -0.39 ± 2.16 p = 1.000 |  |  |
| 18 | 0.50 ± 1.16 p = 0.016 | -0.82 ± 2.48 p = 0.167 | -0.52 ± 2.09 p = 0.812 | -0.28 ± 2.00 p = 1.000 | 0.11 ± 1.25 p = 1.000 |  |

***Supplementary Table 8. Post-hoc pairwise comparison analysis of Short-Form McGill Pain Questionnaire-2 Intermittent Subscale***

*Data is presented as mean difference ± standard deviation (SD) with the later follow up subtracted from the earlier follow up*

| **Follow-up Interval** | **Mean ± SD, *p-*value** | | | | | |
| --- | --- | --- | --- | --- | --- | --- |
|  | **Baseline** | **1-month** | **3-months** | **6-months** | **12-months** | **18-months** |
| Baseline |  |  |  |  |  |  |
| 1 | 1.26 ± 2.20 p < 0.001 |  |  |  |  |  |
| 3 | 1.41 ± 2.21 p = 0.001 | 0.15 ± 1.91 p = 1.000 |  |  |  |  |
| 6 | 0.94 ± 2.15 p = 0.140 | -0.32 ± 2.03 p = 1.000 | -0.46 ± 1.77 p = 0.635 |  |  |  |
| 12 | 0.58 ± 2.01 p = 0.368 | -0.68 ± 2.66 p = 0.727 | -0.82 ± 2.05 p = 0.033 | -0.36 ± 2.02 p = 1.000 |  |  |
| 18 | 0.40 ± 1.42 p = 0.438 | -0.86 ± 2.38 p = 0.087 | -1.01 ± 2.13 p = 0.006 | -0.54 ± 2.20 p = 0.550 | -0.18 ± 1.37 p = 1.000 |  |

***Supplementary Table 9. Post-hoc pairwise comparison analysis of Pain Visual Analogue Scale***

*Data is presented as mean difference ± standard deviation (SD) with the later follow up subtracted from the earlier follow up*

| **Follow-up Interval** | **Mean ± SD, *p-*value** | | | | | |
| --- | --- | --- | --- | --- | --- | --- |
|  | **Baseline** | **1-month** | **3-months** | **6-months** | **12-months** | **18-months** |
| Baseline |  |  |  |  |  |  |
| 1 | 1.11 ± 2.56 p < 0.015 |  |  |  |  |  |
| 3 | 1.18 ± 2.38 p = 0.003 | 0.06 ± 2.74 p = 1.000 |  |  |  |  |
| 6 | 0.87 ± 2.11 p = 0.026 | -0.24 ± 2.68 p = 1.000 | -0.30 ± 2.52 p = 1.000 |  |  |  |
| 12 | 0.52 ± 1.52 p = 0.123 | -0.59 ± 2.91 p = 1.000 | -0.65 ± 2.62 p = 0.800 | -0.35 ± 2.22 p = 1.000 |  |  |
| 18 | 0.37 ± 1.08 p = 0.142 | -0.75 ± 2.62 p = 0.411 | -0.81 ± 2.61 p = 0.252 | -0.51 ± 2.09 p = 0.867 | -0.16 ± 1.51 p = 1.000 |  |

***Supplementary Table 10. Post-hoc pairwise comparison analysis of EQ-5D-5L Index Value***

*Data is presented as mean difference ± standard deviation (SD) with the later follow up subtracted from the earlier follow up*

| **Follow-up Interval** | **Mean ± SD, *p-*value** | | | | | |
| --- | --- | --- | --- | --- | --- | --- |
|  | **Baseline** | **1-month** | **3-months** | **6-months** | **12-months** | **18-months** |
| Baseline |  |  |  |  |  |  |
| 1 | -0.22 ± 0.25 p < 0.001 |  |  |  |  |  |
| 3 | -0.19 ± 0.29 p < 0.001 | 0.04 ± 0.26 p = 1.000 |  |  |  |  |
| 6 | -0.13 ± 0.29 p = 0.010 | 0.09 ± 0.26 p = 0.122 | 0.05 ± 0.27 p = 1.000 |  |  |  |
| 12 | -0.10 ± 0.21 p = 0.009 | 0.12 ± 0.29 p = 0.016 | 0.09 ± 0.25 p = 0.149 | 0.03 ± 0.24 p = 1.000 |  |  |
| 18 | -0.09 ± 0.20 p = 0.011 | 0.13 ± 0.28 p = 0.006 | 0.10 ± 0.25 p = 0.062 | 0.04 ± 0.25 p = 1.000 | 0.01 ± 0.11 p = 1.000 |  |

***Supplementary Table 11. Post-hoc pairwise comparison analysis of EQ-5D-5L Usual Activities***

*Data is presented as mean difference ± standard deviation (SD) with the later follow up subtracted from the earlier follow up*

| **Follow-up Interval** | **Mean ± SD, *p-*value** | | | | | |
| --- | --- | --- | --- | --- | --- | --- |
|  | **Baseline** | **1-month** | **3-months** | **6-months** | **12-months** | **18-months** |
| Baseline |  |  |  |  |  |  |
| 1 | 0.56 ± 0.84 p < 0.001 |  |  |  |  |  |
| 3 | 0.48 ± 1.10 p = 0.017 | -0.80 ± 1.10 p = 1.000 |  |  |  |  |
| 6 | 0.35 ± 1.13 p = 0.247 | -0.21 ± 0.88 p = 1.000 | -0.13 ± 1.06 p = 1.000 |  |  |  |
| 12 | 0.22 ± 0.81 p = 0.505 | -0.33 ± 0.94 p = 0.093 | -0.25 ± 1.06 p = 0.935 | -0.13 ± 1.04 p = 1.000 |  |  |
| 18 | 0.25 ± 0.76 p = 0.154 | -0.30 ± 0.94 p = 0.207 | -0.22 ± 1.09 p = 1.000 | -0.10 ± 1.07 p = 1.000 | 0.03 ± 0.62 p = 1.000 |  |

***Supplementary Table 12. Post-hoc pairwise comparison analysis of EQ-5D-5L Pain and Discomfort***

*Data is presented as mean difference ± standard deviation (SD) with the later follow up subtracted from the earlier follow up*

| **Follow-up Interval** | **Mean ± SD, *p-*value** | | | | | |
| --- | --- | --- | --- | --- | --- | --- |
|  | **Baseline** | **1-month** | **3-months** | **6-months** | **12-months** | **18-months** |
| Baseline |  |  |  |  |  |  |
| 1 | 0.91 ± 1.09 p < 0.001 |  |  |  |  |  |
| 3 | 0.86 ± 1.06 p < 0.001 | -0.05 ± 0.98 p = 1.000 |  |  |  |  |
| 6 | 0.86 ± 1.10  p < 0.001 | -0.05 ± 0.87 p = 1.000 | 0.00 ± 0.84 p = 1.000 |  |  |  |
| 12 | 0.76 ± 1.02 p < 0.001 | -0.14 ± 0.93 p = 1.000 | -0.10 ± 0.79 p = 1.000 | -0.10 ± 0.59 p = 1.000 |  |  |
| 18 | 0.86 ± 1.06 p < 0.001 | -0.05 ± 0.99 p = 1.000 | 0.00 ± 0.79 p = 1.000 | 0.00 ± 0.70 p = 1.000 | 0.10 ± 0.47 p = 1.000 |  |

***Supplementary Table 13. Post-hoc pairwise comparison analysis of EQ-5D-5L Anxiety and Depression***

*Data is presented as mean difference ± standard deviation (SD) with the later follow up subtracted from the earlier follow up*

| **Follow-up Interval** | **Mean ± SD, *p-*value** | | | | | |
| --- | --- | --- | --- | --- | --- | --- |
|  | **Baseline** | **1-month** | **3-months** | **6-months** | **12-months** | **18-months** |
| Baseline |  |  |  |  |  |  |
| 1 | 0.44 ± 0.96 p = 0.008 |  |  |  |  |  |
| 3 | 0.43 ± 0.93 p = 0.008 | -0.10 ± 0.81 p = 1.000 |  |  |  |  |
| 6 | 0.33 ± 0.90  p = 0.068 | -0.11 ± 0.90 p = 1.000 | -0.10 ± 0.71 p = 1.000 |  |  |  |
| 12 | 0.21 ± 0.70  p = 0.336 | -0.12 ± 0.91 p = 0.632 | -0.22 ± 0.81 p = 0.505 | -0.13 ± 0.83 p = 1.000 |  |  |
| 18 | 0.16 ± 0.57 p = 0.478 | -0.11 ± 0.89 p = 0.196 | -0.27 ± 0.85 p = 0.209 | -0.18 ± 0.82 p = 1.000 | -0.05 ± 0.63 p = 1.000 |  |

***Supplementary Table 14. Post-hoc pairwise comparison analysis of Generalised Anxiety Disorder-7***

*Data is presented as mean difference ± standard deviation (SD) with the later follow up subtracted from the earlier follow up*

| **Follow-up Interval** | **Mean ± SD, *p-*value** | | | | | |
| --- | --- | --- | --- | --- | --- | --- |
|  | **Baseline** | **1-month** | **3-months** | **6-months** | **12-months** | **18-months** |
| Baseline |  |  |  |  |  |  |
| 1 | 2.25 ± 4.24 p < 0.001 |  |  |  |  |  |
| 3 | 1.76 ± 4.07 p = 0.016 | -0.49 ± 4.11 p = 1.000 |  |  |  |  |
| 6 | 1.57 ± 4.13  p = 0.055 | -0.68 ± 3.75 p = 1.000 | -0.19 ± 3.28 p = 1.000 |  |  |  |
| 12 | 0.91 ± 3.45  p = 0.622 | -1.35 ± 4.21 p = 0.201 | -0.86 ± 3.27 p = 0.626 | -0.67 ± 2.76 p = 0.904 |  |  |
| 18 | 1.13 ± 3.39 p = 0.156 | -0.13 ± 4.02 p = 0.442 | -0.64 ± 4.01 p = 1.000 | -0.44 ± 3.07 p = 1.000 | -0.22 ± 2.62 p = 1.000 |  |

***Supplementary Table 15. Post-hoc pairwise comparison analysis of Single-Item Sleep Quality Scale***

*Data is presented as mean difference ± standard deviation (SD) with the later follow up subtracted from the earlier follow up*

| **Follow-up Interval** | **Mean ± SD, *p-*value** | | | | | |
| --- | --- | --- | --- | --- | --- | --- |
|  | **Baseline** | **1-month** | **3-months** | **6-months** | **12-months** | **18-months** |
| Baseline |  |  |  |  |  |  |
| 1 | -1.38 ± 3.03 p = 0.009 |  |  |  |  |  |
| 3 | -0.97 ± 2.94 p = 0.016 | 0.41 ± 2.55 p = 1.000 |  |  |  |  |
| 6 | -0.18 ± 2.64  p = 1.000 | 1.21 ± 2.99 p = 0.033 | 0.79 ± 2.57 p = 0.258 |  |  |  |
| 12 | -0.94 ± 2.38  p = 0.041 | 0.44 ± 2.93 p = 1.000 | 0.03 ± 2.91 p = 1.000 | -0.76 ± 1.70 p = 0.273 |  |  |
| 18 | -0.49 ± 2.00 p = 0.829 | 0.89 ± 2.81 p = 0.221 | 0.48 ± 2.80 p = 1.000 | -0.32 ± 2.69 p = 1.000 | 0.44 ± 2.02 p = 1.000 |  |

***Supplementary Table 16. Post-hoc pairwise comparison analysis of Patient Global Impression of Change***

*Data is presented as mean difference ± standard deviation (SD) with the later follow up subtracted from the earlier follow up*

| **Follow-up Interval** | **Mean ± SD, *p-*value** | | | | | |
| --- | --- | --- | --- | --- | --- | --- |
|  | **Baseline** | **1-month** | **3-months** | **6-months** | **12-months** | **18-months** |
| Baseline |  |  |  |  |  |  |
| 1 |  |  |  |  |  |  |
| 3 |  | -0.41 ± 1.25 p = 0.111 |  |  |  |  |
| 6 |  | -0.33 ± 1.14 p = 0.269 | 0.09 ± 1.11 p = 1.000 |  |  |  |
| 12 |  | -0.38 ± 0.87 p = 0.010 | 0.03 ± 1.14 p = 1.000 | -0.05 ± 0.88 p = 1.000 |  |  |
| 18 |  | -0.22 ± 0.73 p = 0.182 | 0.19 ± 1.32 p = 1.000 | 0.10 ± 1.12 p = 1.000 | 0.16 ± 0.80 p = 1.000 |  |

***Supplementary Table 17. Proportion of minimal clinical important differences (MCID) in Pain visual analogue scale (VAS), Brief Pain Inventory (BPI) Interference and BPI Severity PROMs at each timepoint***

*Minimal clinical important difference (MCID) determined by a reduction of 1 point in PAIN VAS, BPI Interference and BPI Severity Scores Scores presented as (%) of total patients for each month, scores in italics are (%) of patients with completed PROMs for each month.*

| **PROM** | **1 month** | **3 months** | **6 months** | **12 months** | **18 months** |
| --- | --- | --- | --- | --- | --- |
| **Pain VAS** | 37 (58.73%)  *(63.79%)* | 32 (50.79%)  *(62.75%)* | 24 (38.10%)  *(57.14%)* | 17 (26.98%)  *(60.71%)* | 11 (17.46%)  *(61.11%)* |
| **BPI Interference** | 32 (50.79%)  *(55.17%)* | 28 (44.44%)  *(54.90%)* | 19 (30.16%)  *(45.24%)* | 17 (26.98%)  *(60.71%)* | 12 (19.05%)  *(66.67%)* |
| **BPI Severity** | 27 (42.86%)  *(46.55%)* | 30 (47.62%)  *(58.82%)* | 21 (33.33%)  *(50.00%)* | 17 (26.98%)  *(60.71%)* | 13 (20.63%)  *(72.22%)* |

***Supplementary Table 18. Proportion of moderately important improvements in Pain visual analogue scale (VAS), Brief Pain Inventory (BPI) Interference and BPI Severity PROMs at each timepoint***

*Moderately important improvement determined by a reduction of ≥30% in PAIN VAS, BPI Interference and BPI Severity Scores Scores presented as (%) of total patients for each month, scores in italics are (%) of patients with completed PROMs for each month.*

|  | **1 month** | **3 months** | **6 months** | **12 months** | **18 months** |
| --- | --- | --- | --- | --- | --- |
| **Pain VAS** | 16 (25.40%)  *(27.59%)* | 18 (28.57%)  *(35.29%)* | 16 (25.40%)  *(38.10%)* | 10 (15.87%)  *(35.71%)* | 5 (7.94%)  *(27.78%)* |
| **BPI Interference** | 22 (34.92%)  *(37.93%)* | 20 (31.75%)  *(39.22%)* | 16 (25.40%)  *(38.10%)* | 13 (20.63%)  *(46.43%)* | 9 (14.29%)  *(50.00%)* |
| **BPI Severity** | 18 (28.57%)  *(31.03%)* | 17 (26.98%)  *(33.33%)* | 12 (19.05%)  *(28.57%)* | 9 (14.29%)  *(32.14%)* | 10 (15.87%)  *(55.56%)* |

***Supplementary Table 19. Proportion of substantial improvements in Pain visual analogue scale (VAS), Brief Pain Inventory (BPI) Interference and BPI Severity PROMs at each timepoint***

*Substantial improvement determined by a reduction of ≥50% in PAIN VAS, BPI Interference and BPI Severity Scores. Scores presented as (%) of total patients for each month, scores in italics are (%) of patients with completed PROMs for each month.*

|  | **1 month** | **3 months** | **6 months** | **12 months** | **18 months** |
| --- | --- | --- | --- | --- | --- |
| **PAIN VAS** | 11 (17.46%)  *(18.97%)* | 13 (20.63%)  *(25.49%)* | 9 (14.29%)  *(21.43%)* | 5 (7.94%)  *(17.86%)* | 0 (0.00%)  *(0.00%)* |
| **BPI Interference** | 9 (14.29%)  *(15.52%)* | 9 (14.29%)  *(17.65%)* | 10 (15.87%)  *(23.81%)* | 8 (12.70%)  *(28.57%)* | 7 (11.11%)  *(38.89%)* |
| **BPI Severity** | 10 (15.87%)  *(17.24%)* | 8 (12.70%)  *(15.59%)* | 6 (9.52%)  *(14.29%)* | 3 (4.76%)  *(10.71%)* | 3 (4.76%)  *(37.50%)* |
